# Molecular adsorbent recirculating system (MARS®) and continuous renal replacement therapy for the treatment of paediatric acute liver failure — two-centre retrospective cohort study

**DOI:** 10.1007/s00431-025-06013-y

**Published:** 2025-02-12

**Authors:** Romit Saxena, Emma C. Alexander, Sander Bontemps, Raman Singla, Henkjan J. Verkade, Vincent E. de Meijer, Martin C. J. Kneyber, Akash Deep

**Affiliations:** 1 Paediatric Intensive Care, King’s College Hospital NHS Foundation Trust, Denmark Hill, London, UK; 2 Paediatric Intensive Care Unit, St Mary’s Hospital, Imperial College Healthcare NHS Trust, London, UK; 3 Division of Paediatric Critical Care Medicine, Department of Paediatrics, Beatrix Children’s Hospital, University Medical Center Groningen, University of Groningen, Groningen, the Netherlands; 4 Division of Pediatric Gastroenterology and Hepatology, Department of Paediatrics, Beatrix Children’s Hospital, University Medical Center Groningen, University of Groningen, Groningen, the Netherlands; 5 Division of HPB and Liver Transplantation, Department of Surgery, University Medical Center Groningen, University of Groningen, Groningen, the Netherlands; 6 Critical Care, Anaesthesiology, Peri-Operative & Emergency Medicine (CAPE), University of Groningen, Groningen, the Netherlands; 7 Department of Women and Children’s Health, School of Life Course Sciences, King’s College London, London, SE1 7EH UK

**Keywords:** Extracorporeal therapy, Liver transplantation, Spontaneous recovery, Hyperammonaemia, Paediatrics, Acute liver failure

## Abstract

To describe outcomes of a cohort of patients with paediatric acute liver failure (PALF) treated with either one of two extracorporeal therapies (ECT) — continuous renal replacement therapy (CRRT) and molecular adsorbent recirculatory system (MARS®). Retrospective, observational, cohort study at two European paediatric intensive care units (PICUs) — UK (2006–2017, CRRT) and the Netherlands (2003–2017, MARS® and CRRT). Patients were children (0–18 years) admitted to the PICU with PALF who required CRRT or MARS®. Between each group, we compared baseline characteristics, biochemical parameters at 0 and 24 h after commencing MARS®/CRRT, and clinical outcomes. In total, 95 patients (23 MARS®, 72 CRRT) were included. The median age at admission for the whole cohort was 4.3 years (interquartile range (IQR) 1.0–12.1), and 47/95 (49.5%) of patients had an indeterminate aetiology. A lower proportion of patients in the MARS® group were on inotropes or were ventilated at admission, and they had a lower Pediatric Index of Mortality 3 risk % than the CRRT group (14.5% (7.5–22) vs 20.4% (16.8–26.4), *p* = 0.002). After treatment, there were no significant differences detected between groups in survival with native liver, or overall survival (15/23 (65.2%) for MARS® and 49/72 (68.1%) for CRRT,* p* = 0.998). *Conclusion*: We did not detect a significant difference in clinical outcomes between PALF patients treated with CRRT or MARS®, despite a relatively sicker cohort in the CRRT group. Further high-quality evidence is necessary regarding the role of extracorporeal therapies in PALF, with consideration of clinical outcomes, feasibility, and cost.

**Table:** 

**What is Known:** *• Outcomes for children with paediatric acute liver failure (PALF) have improved in recent years secondary to improved supportive care aimed at avoiding liver transplantation.* *• Extracorporeal therapies, in particular continuous renal replacement therapy (CRRT), are increasingly applied in the management of these children; however few studies have compared outcomes between different extracorporeal therapies*.	
**What is New:** *• In this retrospective study across two centres, outcomes between patients with PALF treated with CRRT were compared to patients treated with MARS®.* *• There was no significant difference in key clinical outcomes between groups, including survival with native liver and overall survival*.

**Supplementary Information:**

The online version contains supplementary material available at 10.1007/s00431-025-06013-y.

## Introduction

Paediatric acute liver failure (PALF) is a rare clinical syndrome which is associated with high morbidity and mortality. However, an increasing trend towards supportive management aimed at supporting spontaneous liver recovery has reduced the proportion of patients requiring liver transplant (LT) in recent years, and improved survival [1]. Data from the United States Pediatric Health Information system has reported an increase in survival with native liver from 15% in the early post-LT era 1985–1993, to 73% as of 2008–2012 [2]. This outcome is attributed to advancements in supportive care, neuromonitoring, and neuroprotection, as well as the use of extracorporeal therapies [3].

Extracorporeal therapies, such as continuous renal replacement therapy (CRRT) for water-soluble toxins and albumin dialysis (molecular adsorbent recirculatory system, MARS®) for protein-bound toxins, have been used to provide stability to these patients as a bridge to LT or spontaneous recovery [4]. An ideal extracorporeal liver assist device should be able to support both the synthetic and detoxification functions of the liver [3, 5]. Most studies on the use of these modalities in patients with acute liver failure are from the adult population, with conflicting results [6–9]. Paediatric studies comparing the efficacy of extracorporeal therapies are lacking. Though CRRT is not strictly an extracorporeal liver support device, it is the most commonly used extracorporeal modality in intensive care units partially due to staff familiarity, and has been shown to be effective in acute liver failure in children [4, 8]. Other modalities like MARS® are liver-specific but are used less frequently in paediatric intensive care units (PICUs).

To evaluate the evidence basis regarding extracorporeal therapies in PALF, we undertook a retrospective, observational, multicentre, cohort study in two European tertiary liver transplant centres, to compare the clinical and biochemical trajectory of patients with PALF treated with CRRT and MARS®. We hypothesised that CRRT and MARS® would be equally effective in supporting PALF patients resulting in no difference in survival with native liver, or overall survival. To our knowledge, this is the first paediatric study to compare the impact of the two most commonly used extracorporeal modalities, CRRT and MARS®, in paediatric patients, presenting with acute liver failure.

## Materials and methods

This study is a retrospective review of routinely, prospectively collected data from patients with PALF receiving extracorporeal therapy with either CRRT or MARS® at two sites in Europe; King’s College Hospital (KCH), London, UK (2006–2017); and University Medical Center Groningen (UMCG), Netherlands (2003–2017). KCH is one of the largest liver transplant centres in Europe performing 45–60 paediatric liver transplants per year of which 10–15% are performed for PALF. In the Netherlands, all paediatric liver transplantation activity is centralized in the UMCG, with 25–30 liver transplants annually. The PICU at the UMCG is a 20-bed mixed medical-surgical PICU. As a retrospective analysis of routinely collected data extracted from clinical systems, ethical approval was not required at the UMCG. At KCH, the project was registered as a service evaluation (2592).

### Extracorporeal therapies

#### CRRT

This modality was used as per unit protocol at both study centres. Indications at both centres were severe hepatic encephalopathy (grades 3–4), ammonia persistently > 150 or > 200 μmol/L, or for refractory severe acidosis, lactataemia, or electrolyte derangements. The attending consultant paediatric intensivist at both centres determined the requirement and modality for CRRT, with consideration of standard renal indications as well as hepatic indications (encephalopathy and lactataemia). UMCG use the Prismaflex System manufactured by Baxter; KCH use Aquarius™ System manufactured by Nikkiso. Anticoagulant for each therapy was used as per each unit’s local policy (prostacyclin at King’s College Hospital, heparin or no anticoagulant, depending on contraindications including severity of coagulopathy, at the UMCG).

#### MARS®

The molecular adsorbent recirculating system (MARS®; Gambro Lundia, Lund, Sweden) is an extracorporeal liver support device (ECLS) that provides detoxification via membranes and adsorbents [10]. This modality was used only at UMCG as either solo therapy or hybrid with CRRT. The decision to start MARS® was a clinical decision made by the attending physician, indicated for patients with severe encephalopathy (grades 3–4). After 2016, CRRT became the preferred modality over MARS® at UMCG due to greater familiarity within the unit and a stronger evidence base; MARS® was reserved exclusively for children with Wilson’s disease, due to greater evidence of benefit in this group [11]. Duration depended on clinical status but was typically 6–8 h per day. Anticoagulation would either be none, or with heparin, monitored with activated clotting time. Both MiniMARS (extracorporeal volume 50 mL plus lines) and regular MARS® kits (extracorporeal volume 138 mL plus lines) were used depending on patient weight. Standard blood flow was 100–150 mL/min (adult-size filters), 70 mL/min (paediatric filters). Degree of predilution, Na, K, HCO3, and ultrafiltration depended on clinical judgement. Results from some patients treated with MARS® in UMCG have been previously published [10] but without comparison to CRRT.

### Patient selection

Paediatric patients (0–18 years of age) presenting with acute liver failure, who were initiated on extracorporeal therapy, were included. PALF was defined according to the PALF study group criteria [12]. There were two main study groups: patients treated with CRRT only, and patients treated with MARS®. Patients treated with MARS® who were also treated with CRRT were included in the MARS® group, only if they received CRRT for < 24 h total. Patients who received both MARS® and CRRT for > 24 h total were excluded. No patients were treated with plasma exchange. Two subgroups were evaluated: patients treated only with MARS®, and patients treated with CRRT for hepatic indications only.

### Outcomes

We collected admission data on haemodynamic parameters, urine output, grade of hepatic encephalopathy (West Haven criteria [13]), and biochemical parameters. We followed centile parameters for mean arterial pressure according to Paediatric Advanced Life Support Guidelines [14]. Key biochemical parameters were also collected at 24 h after of initiation of the relevant extracorporeal therapy (MARS® or CRRT). These values refer to the closest value to 24 h post-MARS® or 24 h post-CRRT according to group classifications. The aetiology of acute liver failure was sub-classified into toxic, infectious, metabolic, ischaemic, infiltrative, and indeterminate. Pediatric Index of Mortality (PIM) 3 risk % was calculated by the study team for each patient as a marker of illness severity [15].

Indications for extracorporeal therapy were determined by review of the clinical notes by a researcher from the institution in question. Adverse device events during the therapy were defined as any undesirable/unexpected clinical event in a patient, due to device-related effect. Clinical outcomes were duration of ventilation, duration of PICU stay, survival with native liver (SNL) (within admission and at 30 days), requirement for liver transplantation (LT), and overall survival (within admission and at 30 days).

### Statistical analyses

Continuous variables were summarised as mean with standard deviation (SD) or median with interquartile range (interquartile ratio (IQR) 25th–75th centiles) according to normality as determined by the Shapiro–Wilk test and categorical data as count (percentage). The Student *t*, Mann–Whitney *U*, Kruskal–Wallis, or Wilcoxon signed-rank tests were used as appropriate to test differences in continuous variables and the *χ*^2^ test or Fisher’s exact test to compare proportions. Kaplan–Meier curves were used to illustrate the distribution of survival and survival with native liver to 30 days, with log-rank comparisons to determine differences in distributions. All statistical analyses were performed with statistical software IBM-SPSS version 29 (IBM Corporation, New York, USA), Microsoft Excel, or R Studio 2023.03.1 + 446. All tests were two-tailed, and *p* < 0.05 was considered statistically significant.

## Results

### Baseline characteristics

**Fig. 1:**
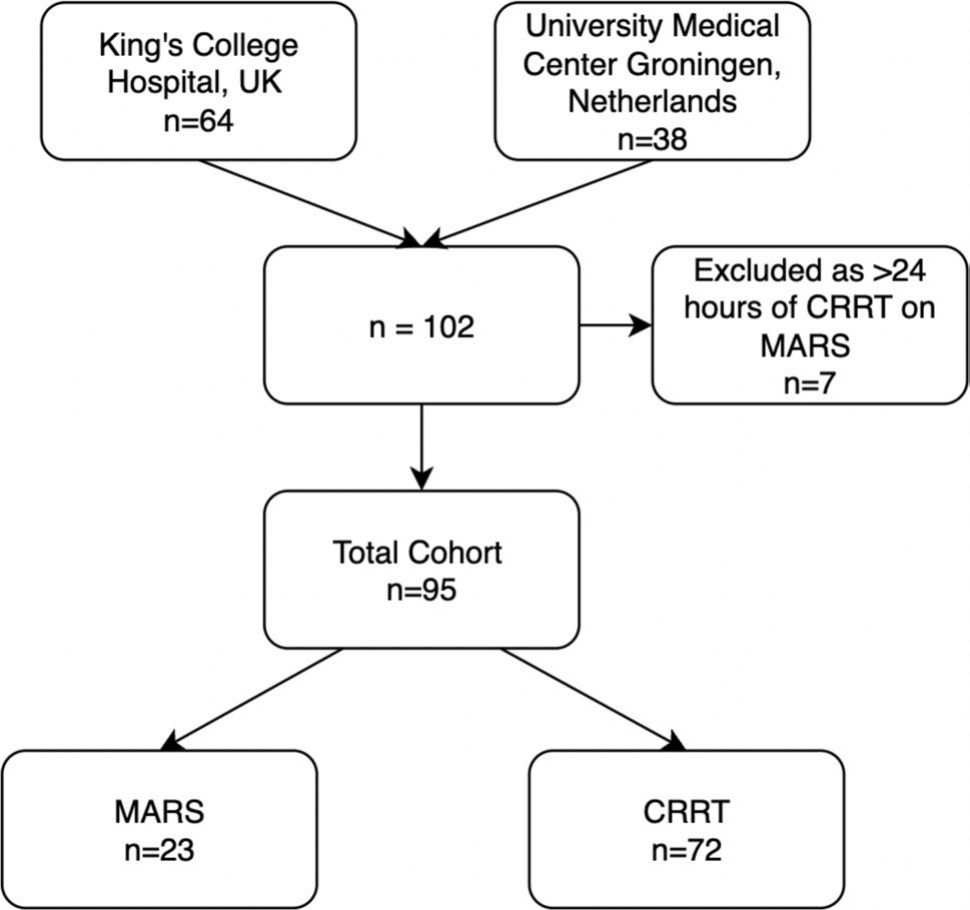
Flow diagram of the study

The final cohort consisted of 95 patients, with 23 patients in the MARS® group, and 72 in CRRT group (Fig. 1). Of the 23 patients in the MARS® group, 15 were treated with MARS® alone, and eight with both MARS® and < 24 h of CRRT (CRRT for a median of 12 h; IQR 10–17 h).

Table 1 describes the baseline characteristics and provides a comparison of clinical-demographic and laboratory data of CRRT and MARS® groups at admission to the PICUs. The median age at admission for the entire cohort was 4.3 years (IQR 1.0–12.1), with median age of 7.8 years (3–14.0) for the MARS® group, and 3.7 years (IQR 0.8–11.7) for the CRRT group (*p* = 0.052). There was a significantly lower mean (± SD) arterial pressure in the CRRT group at admission (mean 67 mmHg (± 15) vs 76 mmHg (± 17), *p* = 0.031); all seven patients with mean arterial pressures below 5th centile for age were in the CRRT group. There was a higher median grade of hepatic encephalopathy at admission in the MARS® group (grade 3 (IQR 2–3) vs grade 2 (IQR 1–3) in the CRRT group, *p* = 0.002). Aetiologies of PALF were similarly represented in each group, and biochemical parameters were similar at admission for both groups. Five patients had Wilson’s disease, three in the CRRT group and two in the MARS® group.

**Table 1:** Baseline characteristics at admission

Parameter	Total cohort (*n* = 95)	MARS® (*n* = 23)	CRRT (*n* = 72)	*p*-value
	(Median (IQR)) or *n* (%)			
Age (in years)	4.3 (1.0–12.1)	7.8 (3–14.0)	3.7 (0.8–11.7)	0.052
Sex (male)	58 (61.1%)	13 (56.5%)	45 (62.5%)	0.790
Weight (in kg)	20 (10.5–40.8)	22.5 (13.3–50)	19.2 (8.4–36.3)	0.099
PIM 3 risk of mortality (%)	19.5 (14.9–25.1)	14.5 (7.5–22)	20.4 (16.8–26.4)	0.002
Hepatic encephalopathy grade	2 (1–3)	3 (2–3)	2 (1–3)	0.002
Urine output (mL/kg/h)	1.3 (0.6–2)	1.2 (0.7–2)	1.3 (0.5–2.0)	0.887
Heart rate (beats per minute)	120 (32)	119 (32)	121 (32)	0.777
Systolic blood pressure (mmHg)	99 (25)	110 (25)	96 (24)	0.022
Mean arterial pressure (mmHg)	69 (16)	76 (17)	67 (15)	0.031
Mean arterial pressure below 5th centile for age	7 (7.4%)	0	7 (9.7%)	0.190
Inotropes/vasopressors at admission	57 (60%)	4 (17.4%)	53 (73.6%)	< 0.001
Ventilated at admission	80 (84.2%)	13 (56.5%)	67 (93.1%)	< 0.001
Aetiology				
Indeterminate	47 (49.5%)	9 (39.1%)	38 (52.8%)	0.368
Toxic	10 (10.5%)	3 (13.0%)	7 (9.7%)	0.700
Infectious	14 (14.7%)	2 (8.7%)	12 (16.7%)	0.506
Metabolic	16 (16.8%)	4 (17.4%)	12 (16.7%)	1.000
Ischemic	1 (1.1%)	0	1 (1.4%)	1.000
Infiltrative	4 (4.2%)	2 (8.7%)	2 (2.8%)	0.246
Autoimmune	3 (3.2%)	3 (13.0%)	0	0.013
Biochemical and haematological baseline parameters				
pH	7.41 (7.31–7.49)	7.42 (7.35–7.49)	7.40 (7.3–7.49)	0.314
PaO2 (kPa)	15.9 (12.5–22.2)	14.6 (11.6–19.5)	16.3 (13.0–22.4)	0.235
FiO2 (%)	0.3 (0.21–0.4)	0.23 (0.21–0.38)	0.3 (0.21–0.4)	0.337
P/F ratio	439 (246–580)	476 (343–547)	417 (243–587)	0.561
PaCO2 (kPa)	4.6 (3.9–5.3)	4.4 (3.7–4.8)	4.8 (4.0–5.5)	0.117
Bicarbonate (mmol/L)	23.1 (19.0–26.6)	22.4 (19.0–23.5)	24 (19.1–27.0)	0.135
Base excess (mmol/L)	–0.1 (–5.8 to 2.0)	–0.8 (–5.6 to 2.2)	–0.1 (–5.9 to 1.9)	0.969
Lactate (mmol/L)	3.5 (2.3–5.6)	3.4 (2.6–5.1)	3.5 (2.1–5.9)	0.945
Sodium (mmol/L)	139 (136–142)	140 (136–142)	139 (136–142)	0.741
Potassium (mmol/L)	3.6 (3.3–4.1)	3.7 (3.1–4.9)	3.6 (3.3–3.9)	0.520
Urea (mmol/L)	2.9 (1.7–7.2)	2.7 (1.5–7.6)	2.9 (1.9–6.6)	0.754
Creatinine (µmol/L)	45 (30–66)	46 (31–71)	45 (28–65)	0.855
AST (U/L)	1444 (530–4661)	1444 (757–5539)	1444 (424–4119)	0.498
Total bilirubin (μmol/L)	235 (91–404)	231 (68–546)	239 (103–392)	0.611
Total white cell count (× 10^9^/L)	7.9 (4.8–12.6)	9 (3.7–15)	7.8 (4.9–11.8)	0.923
Platelets (× 10^9^/L)	156 (73–241)	129 (66–207)	166 (75–245)	0.357

*MARS®,* molecular adsorbent recirculating system; *CRRT*, continuous renal replacement therapy; *IQR*, interquartile range; *FiO2*, fraction of inspired oxygen; PaO2, partial pressure of oxygen; *P/F ratio*, PaO2/FiO2 ratio; *PaCO2*, partial pressure of carbon dioxide; *AST*, aspartate aminotransferase

A significantly higher proportion of patients in the CRRT group were on inotropes/vasopressors at admission (53/72 (73.6%) vs 4/23 (17.4%), *p* < 0.001) and a significantly higher proportion were ventilated at admission (67/72 (93.1%) vs 13/23 (56.5%), *p* < 0.001). Patients in the CRRT group had a higher severity of illness at admission as assessed by PIM 3 risk % (median 20.4 (16.8–26.4) in CRRT group vs 14.5 (7.5–22) in MARS® group, *p* = 0.002).

### Course during PICU stay

Table 2 summarises the clinical course of the extracorporeal therapies. Twenty-three patients were treated with MARS® and 72 patients were treated with CRRT only. Patients most commonly were commenced on MARS® or CRRT within the first day of admission to PICU, at a median of 18 h (IQR 12–23) after admission for the MARS® group, and 14 h (IQR 6–27) after admission for the CRRT group (*p* = 0.358). The median duration of each respective extracorporeal therapy was 8 h (IQR 7.5–15.5) for the MARS® group, and 72 h (IQR 41–168) for the CRRT group.

**Table 2:** Timing, anticoagulation, indications, adverse device events, and outcomes of MARS® vs CRRT groups

Parameter	Total cohort (*n* = 95)	MARS® (*n* = 23)	CRRT (*n* = 72)	*p*-value
Time to initiation of extracorporeal therapy after admission to PICU (hours) (median (IQR))	14 (6–26)	18 (12–23)	14 (6–27)	0.358
Time on CRRT (hours) (median (IQR))	67 (36–150)	12 (10–17)	72 (41–168)	< 0.001
Time on MARS® (hours) (median (IQR))	/	8 (7.5–15.5)	/	/
Anticoagulation used — *n* (%)				
Prostacyclin	44 (58.7%)	0 (0%)	44 (84.6%)	< 0.001
Heparin	23 (30.7%)	17 (73.9%)	6 (11.5%)	< 0.001
No anticoagulation	8 (10.7%)	6 (26.1%)	2 (3.8%)	0.009
No data	20	0	20	
Indications for CRRT/MARS® — *n* (%)				
- Oliguria	30 (31.6%)	5 (21.7%)	25 (34.7%)	0.364
- Hyperkalaemia	6 (6.3%)	3 (13.0%)	3 (4.2%)	0.151
- Fluid overload	9 (9.5%)	1 (4.3%)	8 (11.1%)	0.447
- Hyperammonaemia	23 (24.2%)	9 (39.1%)	14 (19.4%)	0.101
- Encephalopathy	69 (72.6%)	21 (91.3%)	48 (66.7%)	0.041
- Lactataemia	20 (21.1%)	1 (4.3%)	19 (26.4%)	0.036
- Metabolic acidosis	9 (9.5%)	0 (0%)	9 (12.5%)	0.108
Adverse device events while on extracorporeal therapy — *n* (%)				
Major bleeding	6 (10.3%)	3 (13.0%)	3 (8.6%)	0.673
Hypotension necessitating bolus/inotropes	25 (43.1%)	18 (78.3%)	7 (20.0%)	< 0.001
Thrombocytopenia necessitating transfusion	2 (3.4%)	1 (4.3%)	1 (2.9%)	1.000
Other complications	5 (8.6%)	3 (13.0%)	2 (5.7%)	0.376
Missing data	37	0	37	
Total duration of ventilation (hours) (median (IQR))	156 (80–264)	202 (130–324)	128 (64–237)	0.041
Hospital length of stay (in days) (median (IQR))	27 (12–46)	26 (9–49)	27 (13–45)	0.674
Survival with native liver (*n*, %)	18 (18.9%)	4 (17.4%)	14 (19.4%)	1.000
Received liver transplant (*n*, %)	52 (54.7%)	13 (56.5%)	39 (54.2%)	0.966
Time to transplant after PICU admission (days) (median (IQR))	3 (2–4)	3 (2.5–4.5)	3 (2–4)	0.449
Time to transplant after extracorporeal therapy initiation (days) (median (IQR))	2 (2–3.25)	2 (2–3)	3 (1.5–4)	0.323
Overall survival (*n*, %)	64 (67.4%)	15 (65.2%)	49 (68.1%)	0.998

*MARS®*, molecular adsorbent recirculating system; *CRRT*, continuous renal replacement therapy; *IQR*, interquartile range; PICU, paediatric intensive care unit

The most common indication for commencing extracorporeal therapy for the whole cohort was encephalopathy (69/95 (72.6%)). A significantly higher proportion of patients in the MARS® group were commenced on MARS® for encephalopathy than the CRRT group (21/23 (91.3%) vs 48/72 (66.7%), *p* = 0.041). In this study, most patients were anticoagulated as per unit policy, and of the MARS® group, 17/23 (73.9%) were anticoagulated with heparin versus 6/52 (11.5%) (missing data for 20 patients regarding anticoagulation) in the CRRT group (*p* < 0.001). The most common anticoagulant in the CRRT group was prostacyclin (44/52 (84.6%), all from KCH). A small proportion of patients (8/75 (10.7%)) were not anticoagulated.

### Impact of extracorporeal therapy on biochemical parameters

At baseline on commencement of each extracorporeal therapy, the whole cohort had high levels of ammonia (median 134 μmol/L (IQR 92–199)), elevated lactate, serum total bilirubin, and INR (3.9 (IQR 3.1–5.2)) (Table 3). Baseline ammonia levels were significantly higher in the MARS® group than in the CRRT group (173 μmol/L (IQR 106–233) vs 124 μmol/L (IQR 90–175), *p* = 0.046). We identified that there was a significantly greater reduction in median serum lactate between 0 and 24 h of treatment in the CRRT group compared to the MARS® group (–0.8 mmol/L (IQR –1.7 to 0.3) vs 0.2 mmol/L (IQR –0.4 to 1.1), *p* = 0.008) and in a significantly greater reduction in median creatinine in the MARS® group versus the CRRT group (–11 µmol/L (–24 to –5) for MARS® vs –2 µmol/L (–17 to 8) for CRRT, *p* = 0.009). Otherwise, there was no significant difference for any biochemical parameter at baseline, at 24 h, or in assessing the change at 24 h from baseline, between groups. Subgroup analyses including patients treated only with MARS®, and a comparison of the MARS® cohort and patients who received CRRT for hepatic indications, are available in Supplementary Table 1. There was no significant difference in ammonia clearance between the MARS® group and the CRRT hepatic indication subgroup.

**Table 3:** Impact of extracorporeal therapy on biochemical parameters at time 0 and 24 h after starting extracorporeal therapy (MARS® or CRRT)

Parameter	Total cohort (*n* = 95)	MARS® (*n* = 23)	CRRT (*n* = 72)	*p*-value
	Median (interquartile range)			
Ammonia (0 h) (μmol/L)	134 (92–199)	173 (106–233)	124 (90–175)	0.046
Ammonia (24 h) (μmol/L)	105 (79–150)	112 (77–154)	105 (79–150)	1.000
ΔAmmonia_24-0 h_ (μmol/L)	− 23 (− 71 to 5)	− 49 (− 87 to − 1)	− 17 (− 47 to 13)	0.058
Lactate (mmol/L) 0 h	3.5 (2.2–6.1)	3.6 (2.1–5.0)	3.3 (2.2–6.3)	0.862
Lactate (mmol/L) 24 h	2.9 (1.8–5.2)	3.6 (2.4–5.3)	2.8 (1.6–5.1)	0.266
Δ Lactate_24-0 h_ (mmol/L)	− 0.5 (− 1.6 to 0.5)	0.2 (− 0.4 to 1.1)	− 0.8 (− 1.7 to 0.3)	0.008
Creatinine (0 h) µmol/L	45 (33–71)	51 (34–75)	44 (33–64)	0.569
Creatinine (24 h) µmol/L	45 (30–66)	38 (26–56)	49 (31–67)	0.256
Δ Creatinine_24-0 h_ µmol/L	− 6 (− 22 to 3)	− 11 (− 24 to −5)	− 2 (− 17 to 8)	0.009
Bilirubin 0 h µmol/L	219 (91–397)	314 (90–447)	213 (91–358)	0.266
Bilirubin 24 h µmol/L	188 (81–332)	210 (93–360)	185 (76–309)	0.325
Δ Bilirubin_24-0 h_ µmol/L	− 17 (− 60 to − 2)	− 21 (− 102 to − 4)	− 17 (− 53 to − 1)	0.275
INR 0 h	3.9 (3.1–5.2)	3.6 (2.9–4.9)	4.0 (3.2–5.3)	0.326
INR 24 h	3.8 (2.7–5.3)	3.8 (2.6–7.7)	3.8 (2.7–5.3)	0.950
ΔINR_24-0 Hrs_	− 0.0 (− 0.9 to 1.1)	0.1 (− 0.7 to 1.4)	− 0.1 (− 1.2 to 1.0)	0.275

Measured from start of extracorporeal therapy

*MARS®,* molecular adsorbent recirculating system; *CRRT*, continuous renal replacement therapy; *INR*, international normalized ratio

### Adverse device events

Adverse device event data are available in Table 2. A higher proportion of patients in the MARS® group experienced hypotension necessitating a bolus of intravenous fluids or (increased) administration of inotropes (18/23 (78.3%) vs 7/35 (20.0%), *p* < 0.001). There was no difference in any other adverse event outcome.

### Clinical outcomes

The MARS® group had a significantly longer total median duration of ventilation (202 h (IQR 130–324) vs 128 h (IQR 64–237) for the CRRT group, *p* = 0.041). Overall, a relatively low proportion of the cohort survived with native liver (18/95 (18.9%), *p* = 1.000 between groups). This was driven by a high proportion of the cohort who received a liver transplant (52/95 (54.7%), *p* = 0.966 between groups). By the point of hospital discharge, there was no significant difference in overall survival between groups (15/23 (65.2%) for MARS® and 49/72 (68.1%) for CRRT, *p* = 0.998).

**Fig. 2:**
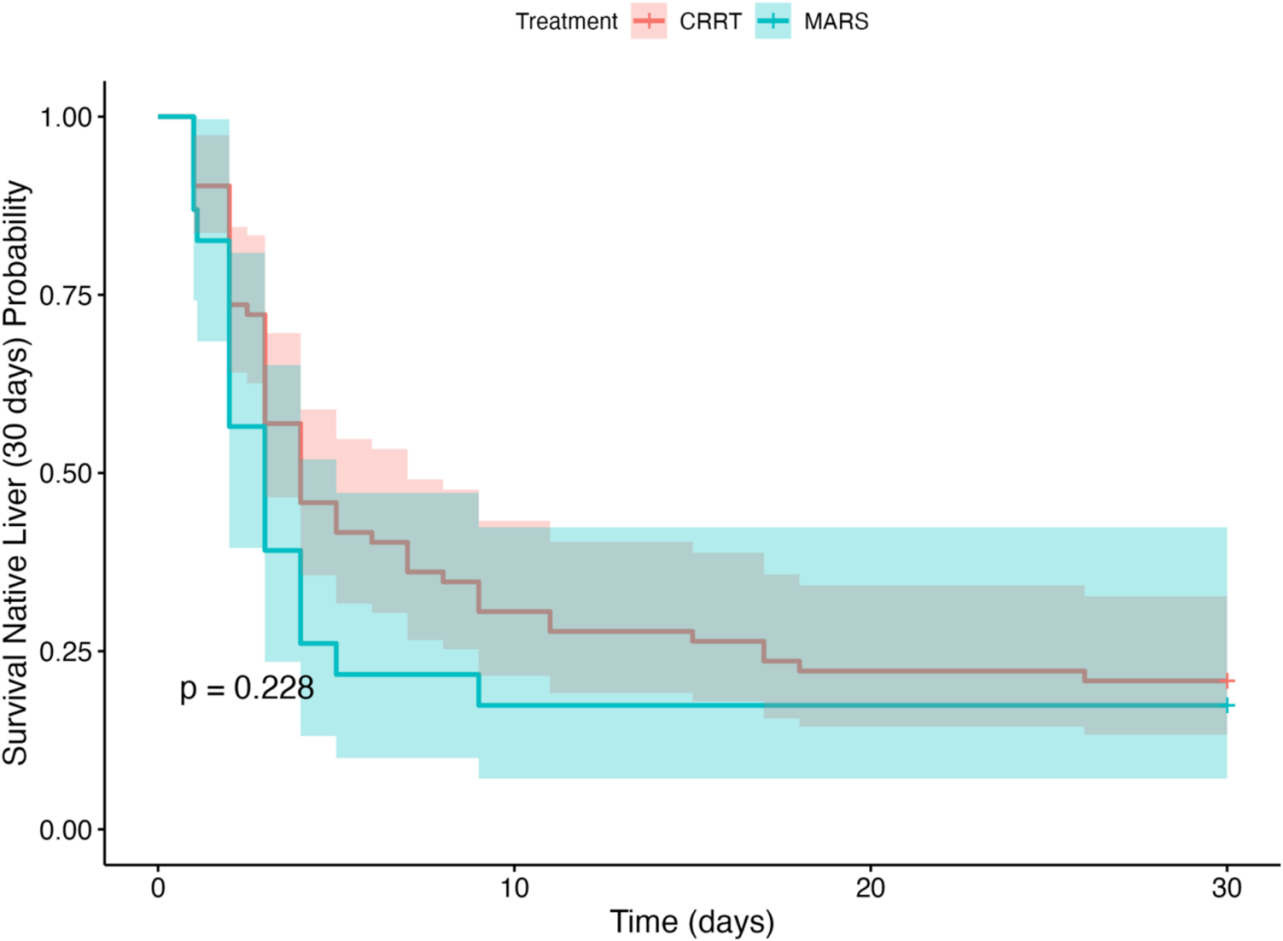
Kaplan–Meier curve of survival with native liver to 30 days, stratified by treatment group (CRRT and MARS®); with log-rank comparison *p* = 0.228; shading indicates 95% confidence interval

**Fig. 3:**
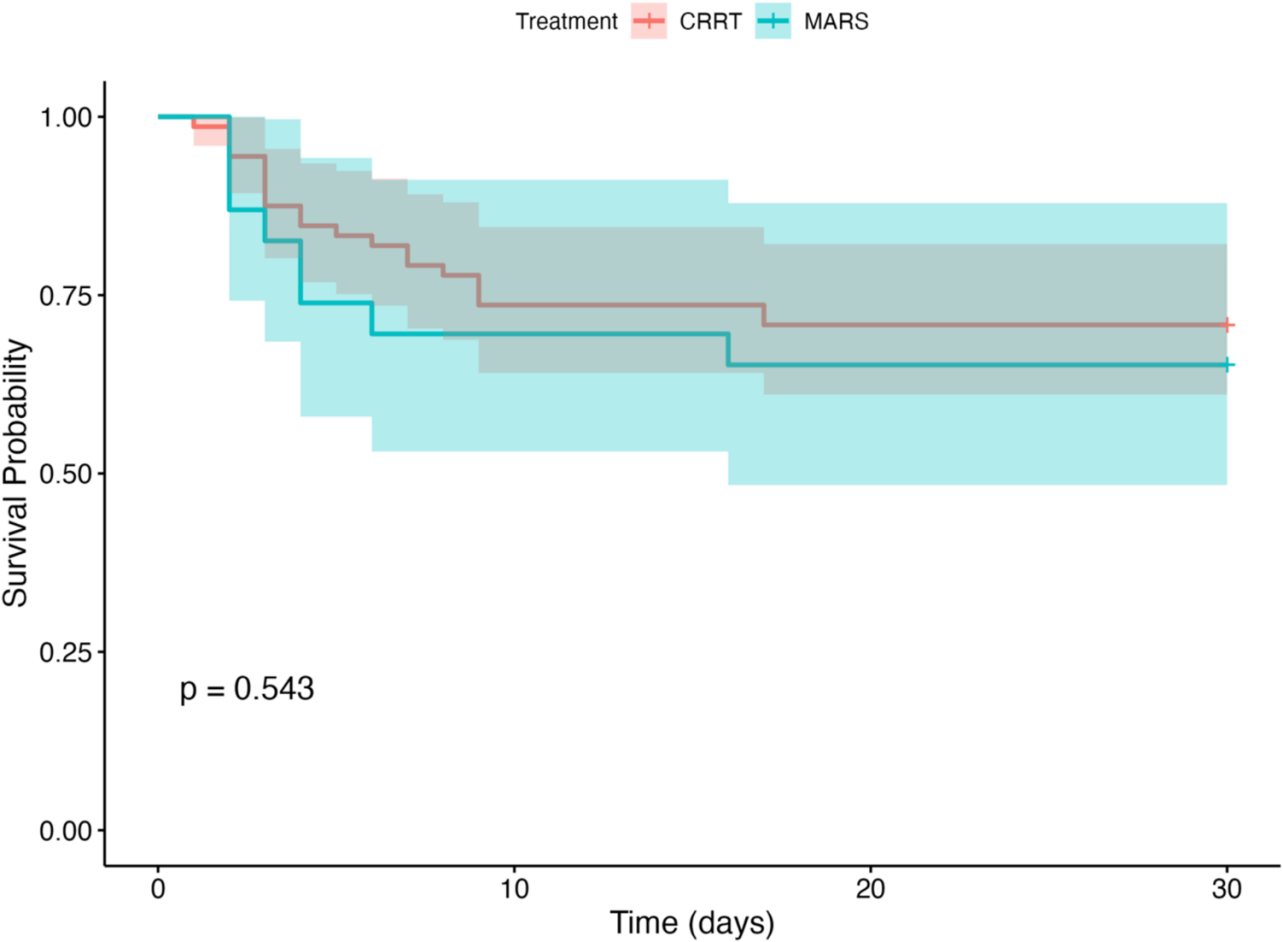
Kaplan–Meier curve of survival to 30 days, stratified by treatment group (CRRT and MARS®); with log-rank comparison *p* = 0.543; shading indicates 95% confidence interval

After analysing survival and survival with native liver up to 30 days after initial PICU admission, there was no significant difference between groups in distribution of survival with native liver to 30 days (Fig. 2, *p* = 0.228) or in distribution of overall survival distribution to 30 days (Fig. 3, *p* = 0.543).

## Discussion

In this retrospective, observational cohort study, including data from two centres, we have described the outcomes from the two most commonly used extracorporeal therapies in PALF – CRRT and MARS®. To our knowledge, this is the first study to directly compare these two modalities in PALF.

Key among our findings is the observation that we did not detect a significant difference in relevant clinical outcomes between the MARS® and CRRT groups. A low proportion of patients in both groups survived with native liver and a relatively low proportion in both groups survived overall (65.2% in the MARS® group, 68.1% in the CRRT group), which may to some extent reflect the advancements of care which have occurred since the first patient was treated. There were some important differences between the groups at baseline, most notably in the PIM 3 risk of mortality %, which was lower in the MARS® group. Additionally, a lower proportion of patients in the MARS® group were admitted to the PICU on inotropes/vasopressors, or were ventilated.

It is unclear why we detected lower illness severity scores, and lower levels of organ support, at baseline in the MARS® group compared to the CRRT group. There may be a degree of confounding by indication, and clinicians having greater confidence in the use of CRRT in a sick population. However, the PICU from the UMCG in the Netherlands has extensive experience with using both MARS® and CRRT. Also, the inclusion of patients in the CRRT group from KCH, which did not use MARS®, to some degree reduces the impact of confounding by indication. PALF patients in the MARS® group trended to be older (median 7.8 years vs 3.7 years). In younger children, especially neonates and infants, detection of important signs and symptoms of acute liver failure can be more challenging than in older children, especially encephalopathy. Babies may be sleepy or have reduced feeding, while behavioural change in older children may be easier to identify, and grade of hepatic encephalopathy easier to classify [16]. Sedation and ventilation also hinder assessment of encephalopathy. This may partially explain the difference in grade of hepatic encephalopathy observed between groups at baseline. Possibly, patients in the CRRT group may have attended later in the course of their illness, and were more unwell, as shown by greater PIM 3 scores and organ support requirements.

Previous studies of MARS® in paediatrics are mostly small case series, but have reported improvements in biochemical parameters (Table 4). Among key paediatric studies, the Texas group published on the use of a hybrid extracorporeal treatment protocol for the treatment of children with ALF or ACLF. This protocol combines use of CRRT for hyperammonaemia with therapeutic plasma exchange for coagulopathy, and MARS® for treatment of hepatic encephalopathy [17]. In 2018 they described the treatment of 15 children with this protocol, with overall survival to hospital discharge of 73% after a median of 6 (IQR 4–7) MARS® treatments. This study provided important data regarding the feasibility of MARS® by an experienced team.

**Table 4:** Case series and cohort studies describing use of MARS® in Paediatric Acute Liver Failure since 2010

Study	Setting	Design	Outcomes
Schaefer et al. [21]	Single centre in Heidelberg, Germany (2002–2010)	Retrospective observational study of 10 children with PALF or acute-on-chronic liver failure (ACLF) treated with MARS® or MARS-mini, with 8 children alternated with plasma exchange and haemodialysis	2/10 survived with native liver 3/10 transplanted and survived 5/10 died Plasma exchange/haemodialysis tended to induce a greater improvement in biochemical parameters
Bourgoin et al. [22]	Single centre in Montreal, Canada (2009–2012)	Case series of 6 children with PALF treated with MARS®	1/6 survived with native liver 2/6 were transplanted and survived 3/6 died without transplant Significant improvement in serum ammonia; some impact on haemodynamics for smaller infants
Rustom et al. [23]	Single centre in Lyon, France (2004–2009)	Case series of 4 children with PALF secondary to Wilson’s disease treated with MARS®	4/4 patients transplanted and survived
Lexmond et al. [10]	Single centre in Groningen, the Netherlands (2002–2011)	Retrospective observational study of 20 children with (17 with PALF) treated with MARS®	In overall cohort of 20: 1/20 survived with native liver 3/20 died without transplant 7/20 died within 3 months of listing MARS® was associated with a significant improvement in biochemical parameters
Akcan Arikan et al. [17]	Single centre in Texas, USA, over 24 months	Retrospective observational study of 15 children with ALF or ACLF treated with a hybrid combination of extracorporeal therapies (CRRT, plasma exchange, and MARS®)	2/15 survived with native liver 9/15 were transplanted and survived 4/15 died without transplant Almost all patients experienced improvement in grade of hepatic encephalopathy (13/15) after treatment Patients received a median of 6 (IQR 4–7) MARS® treatments
Baker et al. [24]	Single centre in New York, USA (2021–2022)	Retrospective observational study of 7 patients (5 children) treated with MARS® for ALF	Of children: 4/5 survived with native liver 1/5 died without transplant MARS® was associated with a significant improvement in biochemical parameters and grade of hepatic encephalopathy
Kaliciński et al. [25]	Single centre in Warsaw, Poland (1990–2022)	Retrospective observational cohort study of 104 children qualified for transplant due to PALF, of which 39 were treated with MARS®	Of patients treated with MARS®: 4/39 survived with native liver 28/39 were transplanted 7/39 died without transplant
This study (2024)	Two PICUs in UK (2006–17) and the Netherlands (2003–2017)	Retrospective, observational study comparing outcomes for 95 children with PALF—23 MARS® (the Netherlands), 72 CRRT (both centres)	MARS® group — overall survival 15/23 (65.2%), survival with native liver 4/23 (17.4%) No significant difference between MARS® and CRRT groups in survival with native liver, or overall survival

The only randomised studies to date have been in adults. The FULMAR trial randomised 49 adult patients with ALF to conventional treatment (including CRRT if required) and 53 patients to receive MARS® alongside conventional treatment (including CRRT if required) [9]. The study did not detect a difference in 6-month survival between groups, which was 75.5% (95% confidence interval (CI), 60.8 to 86.2%) with conventional treatment and 84.9% (CI, 71.9 to 92.8%) in the MARS® group (*p* = 0.28) within the modified intention-to-treat population. The study was limited by the short interval between randomisation and liver transplantation of median 16.2 h, so patients in the MARS® group received a median of only one MARS® treatment session (IQR 1–3). This limitation is also observed in our own study. A second large trial in adults was the RELIEF trial, in adults with acute-on-chronic liver failure [6]. The study did not detect a 28-day transplant-free survival difference (60.7% in the MARS® group vs 58.9%, *p* = 0.79). Renal replacement therapy and liver transplantation were infrequent.

The results from these trials are not necessarily applicable to children. The aetiology of PALF is significantly different from adult ALF, where ALF is most frequently toxin-mediated [18]. The technical difficulty of performing extracorporeal therapies in children is also greater. In the case of RELIEF, acute-on-chronic liver failure has a significantly different pathogenesis and physiology from ALF [19]. Finally, unlike in our study, not all patients in the standard therapy arm were treated with CRRT, limiting these studies’ abilities to make direct comparisons regarding the efficacy of CRRT versus MARS®. These factors increase the urgency of publishing paediatric-specific data on therapeutic outcomes in PALF, especially compared to CRRT.

A final consideration to be made when evaluating MARS® versus CRRT is cost. At KCH, the cost for a 20-kg child receiving CRRT at a dose of 60 mL/kg/h for 72 h including consumables is around £410. In the Netherlands, one session of MARS® Paediatric kit costs €2313 per 24 h. Costs will vary by setting and may be reduced for example when MARS® is used more frequently. However, costs are relevant when guiding treatment selection. Although the results of our study are not conclusive regarding efficacy of CRRT versus MARS®, it should be reiterated that, despite the CRRT group having greater organ support requirements and severity scores, we detected similar outcomes in the MARS® and CRRT groups. Availability and training are also important. A recent survey by the European Society of Paediatric and Neonatal Intensive Care found that most (61%) paediatric intensive care nurses have formal training in CRRT [20]. Lack of training, and variations in practice, would likely be amplified for MARS®.

There are several limitations to this study. The main limitation lies in its retrospective and observational nature. There were several differences between groups at baseline, and there remains residual confounding by indication. Equally, the fact that only one centre used MARS® introduces bias. The group receiving MARS® had some patients who received CRRT as well, although we excluded all patients who received CRRT for over 24 h. Interpretation of results for patients who received MARS® alone without CRRT is challenging because some may have been commenced on CRRT due to non-response to MARS®. The time period for inclusion did not exactly overlap from both centres, with four patients from the UMCG being treated between 2003 and 2005 (one of whom received MARS®). The patients in our cohort generally received MARS® for a short duration. Some variables (including indications) were determined by a researcher reviewing clinical notes, which may have introduced variability. There were missing data for 37/72 patients in the CRRT group regarding adverse device events due to a change in clinical systems. As a high proportion of patients were deeply sedated, we could not assess change in grade of hepatic encephalopathy between groups. There was no long-term follow-up, including of neurological outcomes. Finally, it should be noted that the intensive care supportive management has substantially been improved in recent years [3], and clinical outcomes reported in this cohort may be improved if examining current data.

## Conclusions

In this study, we evaluated outcomes for a group of patients with PALF treated with MARS® for a short duration, and compared them to a group of patients treated with CRRT alone. Our study does not establish the superiority of one modality over the other. However, with consideration of the practicalities (readily available, greater familiarity), lower overall costs, and no detectable difference in survival with native liver or overall survival, we suggest CRRT is preferable as the first-line extracorporeal therapy in children with PALF, while higher-quality evidence is awaited.

## Supplementary Information

Below is the link to the electronic supplementary material.

Supplementary file1 (DOCX 18 KB)

## Data Availability

The data underpinning the findings of this manuscript are available from the corresponding author upon reasonable request.

## Abbreviations

ALF: Acute liver failure
AST: Aspartate aminotransferase
CI: Confidence interval
CRRT: Continuous renal replacement therapy
ECLS: Extracorporeal liver support
HR: Heart rate
INR: International normalised ratio
IQR: Interquartile ratio
KCH: King’s College Hospital
LT: Liver transplantation
MAP: Mean arterial pressure
MARS®: Molecular adsorbent recirculatory system
PALF: Paediatric acute liver failure
PICU: Paediatric intensive care unit
PIM: Pediatric Index of Mortality
SD: Standard deviation
SNL: Survival with native liver
UMCG: University Medical Center Groningen
WBC: White blood cell
