# Supplementary material for: Molecular adsorbent recirculating system (MARS®) and continuous renal replacement therapy for the treatment of paediatric acute liver failure — two-centre retrospective cohort study
Source: Eur J Pediatr. 2025 Feb 12;184(3):192. doi: 10.1007/s00431-025-06013-y (PMC11821745; doi:10.1007/s00431-025-06013-y)
Supplement: Supplementary file 1 — Supplementary file1 (DOCX 18 KB) [file 431_2025_6013_MOESM1_ESM.docx]

| **Supplementary Table 1: Subgroup result for MARS only subgroup, and comparison of MARS outcomes and CRRT outcomes for the subgroup with hepatic indications (encephalopathy and hyperammonaemia)** | | | | |
| --- | --- | --- | --- | --- |
| **Total Cohort (n=95)** | **MARS® only (n=15)** | **MARS® full cohort (n=23)** | **CRRT with hepatic indications (n=56)** | **Comparison between MARS full cohort (n=23) and CRRT cohort with hepatic indications (n=56)** |
| Total duration of ventilation (hours) (Median (IQR)) | 120 (84-228) | 202 (130-324) | 138 (74-217) | 0.031 |
| Hospital length of stay (in days) (Median (IQR)) | 26 (10-46) | 26 (9-49) | 31 (13-54) | 0.453 |
| Survival with native liver (n, %) | 3 (20%) | 4 (17.4%) | 8 (14.3%) | 0.738 |
| Received liver transplant (n, %) | 8 (53.3%) | 13 (56.5%) | 34 (60.7%) | 0.926 |
| Overall survival (n, %) | 10 (66.7%) | 15 (65.2%) | 39 (69.6%) | 0.906 |
| Ammonia (0 Hrs.) (μmol/L) | 207 (121-261) | 173 (106-233) | 145 (93-183) | 0.173 |
| Ammonia (24 hrs) (μmol/L) | 112 (77-156) | 112 (77-154) | 107 (90-157) | 0.609 |
| ΔAmmonia _24-0 hrs_ (μmol/L) | -71 (-101- -30) | -49 (-87--1) | -22 (-55-14) | 0.086 |
| Lactate (mmol/L) 0 hours | 3.8 (3.1-5.0) | 3.6 (2.1-5.0) | 3.4 (2.2-6.3) | 0.787 |
| Lactate (mmol/L) 24 hrs | 3.9 (3.1-5.3) | 3.6 (2.4-5.3) | 2.8 (1.8-5.1) | 0.340 |
| Δ Lactate _24-0 hrs_ (mmol/L) | 0.2 (-0.4-1.1) | 0.2 (-0.4-1.1) | -0.8 (-1.7 – 0.4) | 0.010 |
| Creatinine (0 Hrs) µmol/L | 49 (34-69) | 51 (34-75) | 43 (30-61) | 0.288 |
| Creatinine (24 hrs) µmol/L | 36 (25-49) | 38 (26-56) | 44 (29-62) | 0.575 |
| Δ Creatinine _24-0 hrs_ µmol/L | -11 (-22—7) | -11 (-24--5) | -2 (-9-7) | 0.003 |
| Bilirubin 0 hrs µmol/L | 329 (115-447) | 314 (90-447) | 244 (104-390) | 0.379 |
| Bilirubin 24 hrs µmol/L | 304 (112-360) | 210 (93-360) | 194 (82-322) | 0.582 |
| Δ Bilirubin _24-0 hrs_ µmol/L | -21 (-76-9) | -21 (-102--4) | -14 (-41-2) | 0.157 |
| INR 0 hrs | 3.8 (3.1-5) | 3.6 (2.9-4.9) | 4.3 (3.6-5.3) | 0.135 |
| INR 24 hrs | 4.2 (2.6-10) | 3.8 (2.6-7.7) | 3.9 (2.9-5.2) | 0.795 |
| ΔINR _24-0 Hrs_ | 0.3 (-0.8-2.6) | 0.1 (-0.7-1.4) | -0.1 (-1.2-0.7) | 0.273 |
